# Supplementary material for: The causal relationship between the human gut microbiota and pyogenic arthritis: a Mendelian randomization study
Source: Front Cell Infect Microbiol. 2024 Nov 26;14:1452480. doi: 10.3389/fcimb.2024.1452480 (PMC11629706; doi:10.3389/fcimb.2024.1452480)
Supplement: Supplementary file 1 [file DataSheet1.zip › mendelian test/ebi-a-GCST90027541.csv_leave_one_out.pdf]

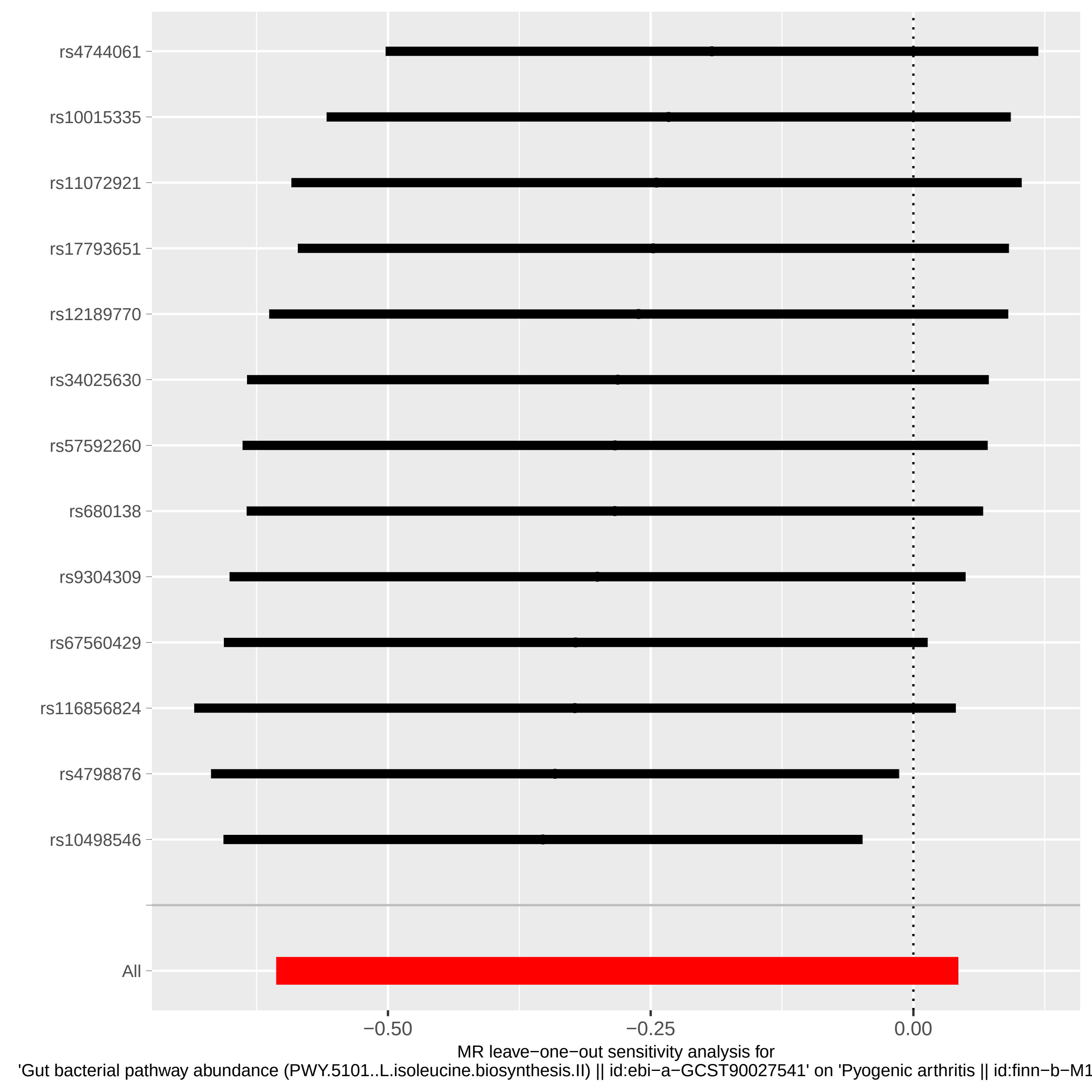

rs4744061
rs10015335
rs11072921
rs17793651
rs12189770
rs34025630
rs57592260
rs680138
rs9304309
rs67560429
rs116856824
rs4798876
rs10498546
All
−0.50
−0.25
0.00
MR leave−one−out sensitivity analysis for
'Gut bacterial pathway abundance (PWY.5101..L.isoleucine.biosynthesis.II) || id:ebi−a−GCST90027541' on 'Pyogenic arthritis || id:finn−b−M1
